# Autophagy: the misty lands of *Chlamydia trachomatis* infection

**DOI:** 10.3389/fcimb.2024.1442995

**Published:** 2024-09-06

**Authors:** Shan Zhang, Yufei Jiang, Yonghui Yu, Xuan Ouyang, Dongsheng Zhou, Yajun Song, Jun Jiao

**Affiliations:** State Key Laboratory of Pathogen and Biosecurity, Academy of Military Medical Sciences, Beijing, China

**Keywords:** *Chlamydia trachomatis*, inclusion, autophagy, mitophagy, LC3

## Abstract

*Chlamydia* are Gram-negative, obligate intracellular bacterial pathogens that infect eukaryotic cells and reside within a host-derived vacuole known as the inclusion. To facilitate intracellular replication, these bacteria must engage in host-pathogen interactions to obtain nutrients and membranes required for the growth of the inclusion, thereby sustaining prolonged bacterial colonization. Autophagy is a highly conserved process that delivers cytoplasmic substrates to the lysosome for degradation. Pathogens have developed strategies to manipulate and/or exploit autophagy to promote their replication and persistence. This review delineates recent advances in elucidating the interplay between *Chlamydia trachomatis* infection and autophagy in recent years, emphasizing the intricate strategies employed by both the *Chlamydia* pathogens and host cells. Gaining a deeper understanding of these interactions could unveil novel strategies for the prevention and treatment of *Chlamydia* infection.

## 1 Introduction

*Chlamydia* are Gram-negative, obligate intracellular bacterial pathogens and symbionts that inhabit a wide range of hosts, from humans to amoebae. The *Chlamydiaceae* family includes at least 13 species that are pathogenic to humans or animals (Bachmann et al., 2014). Some species, like *Chlamydia psittaci* and *Chlamydia abortus*, are known as zoonotic pathogens, while *Chlamydia trachomatis* is endemic to humans, primarily infecting the female genital tract (Jury et al., 2023). Additionally, *Chlamydia pneumoniae* is a prevalent pathogen in both humans and animals, commonly associated with respiratory tract infections (Cheong et al., 2019).

As obligate intracellular bacteria, *Chlamydia* spp. invade eukaryotic cells and reside within a cytoplasmic membrane-bound vacuole known as the inclusion (Stelzner et al., 2023). These bacteria exhibit a biphasic developmental cycle that hinges on specific host-cell interactions for nutrient acquisition necessary for intracellular proliferation (Stephens et al., 1998). The infection process is initiated by the non-replicative, infectious elementary bodies (EBs). Following host cell entry, EBs differentiate into the noninfectious, and replicative reticulate bodies (RBs) (Omsland et al., 2012). Upon completing their developmental cycle, RBs revert to EBs, which are then released upon host cell lysis to propagate subsequent infections. Additionally, some *Chlamydia* spp. can also exit their host by extrusion, without causing cell lysis.

*Chlamydia trachomatis*, a critical species within this genus, is the foremost pathogen causing trachoma and sexually transmitted infection (STI), and will be the focus of this review (Liang et al., 2017). Trachoma is the leading infectious cause of blindness globally, with the World Health Organization (WHO) estimating that approximately 190 million people are at risk of trachoma blindness (Woodhall et al., 2018). Most STIs caused by *C. trachomatis* are asymptomatic, but untreated infections can result in pelvic inflammatory disease (Poston et al., 2019). *C. trachomatis* strains are classified into two major biovars and further subtyped by serovars based on the chlamydial major outer membrane protein (MOMP) (Miyairi et al., 2006). The lymphogranuloma venereum (LGV) biovar (serovars L1, L2, and L3) targets genital tissues and macrophages, causing the invasive STI known as ‘LGV’ (Abdelsamed et al., 2013; Hepler et al., 2018). Among the non-LGV biovar, serovars A-C infect the ocular tissue and cause trachoma. In contrast, serovars D-K primarily cause inflammatory diseases such as pelvic inflammatory disease and tubal factor infertility (Hepler et al., 2018).

## 2 Molecular pathogenesis

As an obligate intracellular organism, *C. trachomatis* employs its virulence factors intricately engage with host cellular mechanisms, eliciting host immune responses and triggering a cascade of cellular pathological alterations (Chen et al., 2019). The Type III secretion system (T3SS) consisting of a needle-like injectosome complex, facilitates the translocation of effector proteins from the chlamydial cytoplasm into the host cell’s cytoplasm and inclusion’s lumen (Deng et al., 2017). It is hypothesized that there are over 80 anti-host T3SS-secreted effectors participating in diverse biological processes, including but not limited to the invasion, cytoskeletal structure reconstruction, modulation of vesicular interactions, apoptosis, signal transduction, promoting the bacterial survival and replication (Betts-Hampikian and Fields, 2010). To date, many proposed effectors remain unverified and even more are yet to be characterized. The advent of genetic manipulation techniques has significantly expanded the research on elucidating the function of T3SS effectors (Wan et al., 2023).

Another important virulence factor is a highly conserved ~7.5 kb plasmid, encoding encodes eight glycoproteins (pGP1-8) that facilitate the ascension of infection, elicit pro-inflammatory responses, and promote extrusion processes (Zhong, 2017; Jury et al., 2023). Under stress conditions, the copy number of *C. trachomatis* plasmid increases, presumably to upregulate T3SS-secreted substrates and ensure *in vivo* survival. Nevertheless, the precise role of chlamydial plasmid in its pathogenesis remains to be fully elucidated.

## 3 Host autophagy in *Chlamydia trachomatis* infection

To date, three different pathways of autophagy have been described: chaperone-mediated autophagy (CMA), macroautophagy, which is the most extensively studied one, and microphagy (Kim and Lee, 2014). Macroautophagy (hereafter referred to as autophagy) is a fundamental and evolutionary conserved progress that facilitates the delivery of dysfunctional organelles or invading pathogens to lysosome degradation, thereby preserving cellular homeostasis in eukaryotic cells (Yu et al., 2018). Analogous to endocytosis, both pathways converge at the lysosome, where their respective cargos are degraded. Endocytosis is crucial for various cellular processes, including nutrient uptake, cell adhesion and migration, pathogen entry, by internalizing extracellular cargos from the plasma membrane. However, autophagy involves the engulfment of cytoplasmic contents (cargos) into a double membrane vesicle known as the autophagosome. This multifaceted process involves autophagy initiation, autophagosome formation, autophagolysosome formation, and cargo degradation (Hansen and Johansen, 2011). Autophagosomes acquire d lysosomal enzymes necessary for degrading the vacuolar contents, which is subsequently released into the cytosol (Yim and Mizushima, 2020).

Typically, autophagy restricts bacterial proliferation and dissemination, thereby mitigating infection severity. Examples of autophagy-targeted bacteria include *Legionella*, *Salmonella* and *Mycobacterium* (Sharma et al., 2018; Thomas et al., 2020). Correspondingly, these bacteria have developed various strategies to form unique parasitophorous vacuoles and evade the autophagic capture (Deretic et al., 2013). For instance, the cysteine protease RavZ from *Legionella pneumophila* strain inhibits autophagy by irreversibly deconjugating the ubiquitin-like autophagy-related protein 8 (ATG8) from autophagosome membranes (Choy et al., 2012). *Salmonella* escaped from *Salmonella*-containing vacuoles (SCV) into the cytosol, where it is initially ubiquitinated, followed by the recruitment of selective autophagy receptors p62/SQSTM1 and OPTN (optineurin), leading to its phagocytosis and degradation (Gomes and Dikic, 2014). IFN-γ induction of autophagy contributed to the control of *Mycobacterium tuberculosis* intracellular proliferation (Kim et al., 2011). The role of IFN-γ in autophagy involves the interferon-induced guanylate-binding protein (GBP), which promotes the oxidative killing and the delivery of antimicrobial peptides to autophagolysosome (Gutierrez et al., 2004). However, unlike the aforementioned pathogens, *Coxiella burnetii* exploits the autophagy for its benefits. The effector protein CvpF of *C. burnetii*, interacts with the host small GTPase RAB26 to stimulate the autophagy flux, promoting the vacuole biogenesis and its virulence (Siadous et al., 2020) Another effector protein, CpeB, targets Rab11a to promote the accumulation of microtubule-associated protein light chain 3-II (LC3-II), a lipidated form of LC3 that is associated with autophagosomal membranes, thus sustaining the bacteria infection and virulence (Fu et al., 2022).

The role of autophagy in C. trachomatis development is ambiguous. Conflicting results have been reported, depending on experimental conditions, chlamydial serovars, and cell lines used (Witkin et al., 2017). For example, no distinct rim of autophagosomal protein-specific staining was observed around the *C. trachomatis*’s inclusions (Al-Younes et al., 2004). And the inhibition of inclusion-lysosomes fusion is beneficial for the intracellular survival of *C. trachomatis* throughout the its developmental cycle, suggesting the evasion of bacteria from the autophagy-mediated destruction (Heinzen et al., 1996). Concurrently, *C. trachomatis* might modulate interactions between the inclusions with host vesicles and promote nutrient acquisitions from the cytoplasm (McClarty, 1994). Nutrient availability significantly impacts the normal development of *C. trachomatis*. Thus, it can be inferred that autophagy may serve as a nutrient source of for the replicating bacteria, as demonstrated by the inhibition of the intracellular replication of *C. trachomatis* serovar L2 upon treatment with autophagy inhibitors (Al-Younes et al., 2004).

## 4 Host autophagy manipulation by *C. trachomatis*

### 4.1 Initiating autophagy induced by *Chlamydia* infection

*Chlamydia trachomatis* has a substantially contracted genome (1.04 Mb encoding 895 open reading frames) that lacks many metabolic enzymes, conferring a restricted biosynthetic capacity that necessitates reliance on eukaryotic host cells for essential metabolites (Stephens et al., 1998). *C. trachomatis* acquires a variety of nutrients, including amino acids, nucleotides and lipids from the lysosomal-mediated degradation of proteins (Bastidas et al., 2013) and various transporters (Damiani et al., 2014). The active intracellular growth of *C. trachomatis* results in significant decreases in the nutrient pools in host cells, thereby triggering autophagy around the midpoint of the *C. trachomatis* developmental cycle, suggesting that *Chlamydia* infection induces autophagy to secure nutrients for bacterial proliferation (Pachikara et al., 2009). Previous studies have demonstrated that *Chlamydia* infection could induce autophagy. Autophagosome-associated markers, including microtubule-associated protein light chain 3 (MAP-LC3, autophagosomal markers) and calreticulin, are redistributed to the inclusions of *C. trachomatis* (Al-Younes et al., 2004). The plasmid-encoded protein pORF5 of *C. trachomatis* activates the unfolded protein response (UPR) to induce autophagy, with the MAPK/ERK inhibitor PD98059 partially attenuating pORF5-induced autophagy, indicating that pORF5 activates UPR to induce autophagy via the MAPK/ERK signaling pathway (Wen et al., 2020). The infection of mouse embryo fibroblasts (MEFs) by *C. trachomatis* serovar L2 correlates with upregulated autophagy-related protein production (Pachikara et al., 2009), whereas autophagy is suppressed in p62−silenced *Chlamydia*-infected HeLa229 cell line (Wang et al., 2021). The numbers of autophagosomes and the expression of LC3-II and Beclin1 were increased, and the expression of p62 was downregulated in *C. psittaci-*infected human bronchial epithelial cells (HBEs) (Chen et al., 2022). These findings align with other studies showing that Atg5 silencing significantly inhibits the autophagy in the *Chlamydia*-infected HeLa229 cells (Wang et al., 2019). Furthermore, Atg5 knockdown promotes the secretion of proinflammatory cytokines IL-1β and IFN-γ in *C. trachomatis*-infected cells, suggesting an anti-inflammatory role for Atg5 in the autophagy induction during *chlamydia* infection (Wang et al., 2019).

Additionally, autophagy inhibitors impede the intracellular replication of *Chlamydia*. Hep-2 cells infected with *C. trachomatis* Serovar L2 subsequently treated with 3-methyladenine (3-MA) exhibits a decreased expression LC3-II/LC3-I ratio, abnormal inclusion maturation, and diminished progeny, indicating that 3-MA inhibits of *C. trachomatis* growth by reducing autophagy (Al-Younes et al., 2004). Bafilomycin A1 (BafA1), a vacuolar H+/ATPase inhibitor that blocks lysosomal acidification and functions, is one of the widely used to inhibit the last stage of autophagy and impair the growth of *C. trachomatis* (Ouellette et al., 2011).

### 4.2 Autophagy restricts *Chlamydia* growth

One of the host cell strategies to block intracellular bacterial proliferation involves phagolysosomal degradation, wherein the host cell directs internalized bacteria into vesicles delivered to the acidic lysosome for degradation. Early-stage *C. trachomatis* infected RAW macrophages exhibit impaired intracellular proliferation, while inclusions of *C. trachomatis* were positive for the autophagy marker LC3 during the late stage of infection. Sun et al. also reported that *C. trachomatis* is rapidly transported to lysosomes, with significantly augmented bacterial growth in RAW macrophages pretreated with BafA1 (Sun et al., 2012). These findings underscore the crucial role of lysosomal inclusion fusion in modulating *C. trachomatis* infection.

Interferon γ (IFN-γ), a cytokine involved in immune regulation plays a pivotal role in cell-intrinsic immunity against various intracellular pathogens, including *Chlamydia* (Vollmuth et al., 2022). Exogenous IFN-γ diminished chlamydial infectivity by 50% in wild-type human macrophages. Al-Zeer and colleagues demonstrated that the human GBP1/2 are recruited to bacterial inclusions in human macrophages upon IFN-γ stimulation, prompting the rerouting of the typically nonfusogenic bacterial inclusions for lysosomal degradation. This suggests that autophagy confines *C. trachomati*s proliferation in human macrophages via IFN-γ-inducible GBPs (Al-Zeer et al., 2013).

Subsequent studies identified immunity-related GTPases (IRGs), such as Irga6, as critical in IFN-γ-induced autophagy for *C. trachomatis* eradication (Al-Zeer et al., 2009). Enhanced *C. trachomatis* growth has been observed in autophagy-deficient Atg5^−/−^ or Irga6^−/−^ MEFs upon IFN-γ stimulation compared to that in wild-type MEFs. IFN-γ treatment inhibits the growth of *C. trachomatis* inclusions and formation of infectious EBs via lysosomal fusion with early inclusions, a process reversible with BafA1 treatment (Al-Zeer et al., 2009; Yasir et al., 2011).

Additionally, autophagy pathway component deficiency in MEFs or macrophages leads to increased *C. pneumoniae* proliferation, indicating autophagy’s bactericidal role (Crother et al., 2019). The inflammasome, a crucial innate immune component, fight against pathogenic microorganisms (Biasizzo and Kopitar-Jerala, 2020). Autophagy negatively regulates the nucleotide binding domain and leucine-rich repeat (NLR) pyrin domain containing 3 (NLRP3) inflammasome and subsequent IL-1β secretion (Crother et al., 2019). Recent studies have revealed that *Chlamydia* induces inflammasome priming and activation signals (Webster and Goodall, 2018), and the autophagy deficiency results in increased pulmonary inflammasome activity and IL-1β production in response to *C. pneumoniae* infection in mice (Crother et al., 2019).

### 4.3 Autophagy-independent manner

Autophagy-related gene (ATG) proteins are pivotal in orchestrating the biogenesis of autophagosomes, facilitating the transport of cytoplasmic constituents to lysosomes or vacuoles (Kirkin and Rogov, 2019). Furthermore, ATG protein activities modulate cell cycle progression through both autophagy-dependent and autophagy-independent pathways (Palikaras et al., 2018).

Research indicates that the proliferation of *C. trachomatis* is modulated by ATG9A and ATG16L1 via autophagy-independent mechanisms. Hamaoui et al. revealed that the *Chlamydia* effector protein TaiP (CT622) targets ATG16L, disrupting the ATG16L1-TMEM59 interaction and redirecting Rab6-positive vesicles to the inclusion (Li et al., 2008). Moreover, a recent investigation elucidated that ATG9A organizes ATG9A vesicles to inclusions and mediates Golgi apparatus redistribution to enhance *C. trachomatis* proliferation (Zou et al., 2018).

LC3 proteins are extensively utilized as biomarkers in autophagy flux studies. The cytosolic non-lipidated form of LC3 (LC3-I) conjugates with phosphatidylethanolamine, forming the LC3-phosphatidylethanolamine conjugate (LC3-II), which is subsequently recruited to autophagosomal membranes. Notably, LC3 has been implicated in stabilizing the microtubule network of the cytoskeleton (Lei et al., 2017). Al-Younes et al. demonstrated that LC3 associates with *C. trachomatis* inclusions along microtubules, and depletion of LC3 in autophagy-deficient cells significantly impairs chlamydial propagation, highlighting a novel role for LC3, independent of autophagy, in *Chlamydia* pathogenesis (Rucks, 2023).

### 4.4 *Chlamydia* protects mitochondrial function by mitophagy

Autophagy can be either selective or nonselective. Selective autophagy leads to the lysosomal degradation of specific cargoes like mitochondria, known as mitophagy (Kirkin and Rogov, 2019). Mitophagy is crucial for maintaining mitochondrial quality control and homeostasis, as it facilitates the selective degradation of dysfunctional mitochondria (Palikaras et al., 2018).

pORF5 among the eight plasmid-encoded proteins in *C. trachomatis*, is the sole secreted protein (Li et al., 2008). Previous studies have confirmed that pORF5 modulates the expression of autophagy-related proteins in host cells (Zou et al., 2018). Lei et al. demonstrated that *C. trachomatis* pORF5 preserves mitochondrial function in HeLa cells carbonyl treated with cyanide 3-chlorophenylhydrazone (CCCP). Subsequent experimental data indicated following pORF5 treatment, LC3 accumulated around mitochondria, with p62 subsequently recruited to the mitochondria via its binding to LC3, implying that pORF5 safeguards mitochondrial function through the mechanism of mitophagy (Lei et al., 2017).

## 5 Conclusion and future directions

In summary, *C. trachomatis* has developed a variety of strategies to exploit host cellular mechanisms, thereby facilitating its own intracellular proliferation Autophagy, the host cell’s intrinsic defense mechanism, presents a dual role for *C. trachomatis*. On one hand, *C. trachomatis* can induce autophagy to procure nutrients sustain host cell homeostasis. On the other hand, the fusion of autophagosomes and lysosomes leads to acidification and the activation of degradative enzymes that inhibit the proliferation of *C. trachomatis* (**Table 1**). However, the exact interplay between C. trachomatis and autophagy remains to be elucidated.

**Table 1:** Host autophagy manipulation by *Chlamydia trachomatis*.

Host autophagy manipulation by *Chlamydia trachomatis*	*Chlamydia*-Related proteins	Host-related proteins	Roles in *Chlamydia* infection	References
Initiation of Autophagy				
	pORF5		Actives UPR to induce autophagy via the MAPK/ERK signaling pathway	(Wen et al., 2020)
		p62	The level of autophagy was suppressed in p62–silencing *Chlamydia*-infected HeLa229 cell line	(Wang et al., 2021)
		ATG5	Regulates the level of autophagy and anti-inflammatory	(Wang et al., 2019)
		Incubation of 3-MA	Blocks inclusion maturation	(Al-Younes et al., 2004)
		Incubation of BafA1	Blocks lysosomal acidfication functions	(Ouellette et al., 2011)
Autophagy Restricts *Chlamydia* Growth				
		GBP1/2	GBP1/2 were recruited to bacterial inclusions in human macrophages upon stimulation with IFN-γ, and triggers rerouting of the typically nonfusogenic bacterial inclusions for lysosomal degradation	(Al-Zeer et al., 2013)
		Irga6	*Chlamydia* growth was enhanced in autophagy-deficient Irga6^−/−^ MEFs by IFN-γ stimulation	(Al-Zeer et al., 2009; Yasir et al., 2011)
Autophagy Independent Manner				
	Taip	ATG16L1	Targets ATG16L1 and thus disrupts ATG16L1-TMEM59 interaction and rerouts Rab6-positive vesicles toward the inclusion	(Li et al., 2008)
		ATG9A	Arranges ATG9A vesicles to inclusions and involves Golgi redistribution	(Lei et al., 2017)
		LC3	Interacts with the *Chlamydia* inclusion along microtubules for the stabilization of microtubular network	(Rucks, 2023)
Mitophagy				
	pORF5		Protects mitochondrial function	(Lei et al., 2017)

*Coxiella burnetii*, an obligate intracellular bacterial pathogen, can actively manipulate the autophagic pathway to establish an intracellular niche known as the *Coxiella*-containing vacuole (CCV). Accumulating evidence indicates that the functional type IV secretion system (T4SS) of *C. burnetii* is pivotal for the manipulation of host autophagy. Similar to *C. burnetii*, *Chlamydia* survives and reproduces within the inclusions that sequester the pathogens with the cytoplasma and can manipulate host cellular processes through the T3SS-secreted effector proteins. Currently, research on C. trachomatis T3SS-secreted effector proteins is limited; many proposed effectors are yet to be confirmed, and even more remain uncharacterized, with only evidence of secretion predominantly derived from heterologous T3SS expression systems (Rucks, 2023). Genetic approaches have made substantial advancements in *C. trachomatis*, which should be leveraged to further dissect on autophagy (Bastidas and Valdivia, 2023). Ultimately, a deeper understanding of the molecular pathogenesis of *C. trachomatis* will be of great help for increasing the diagnostic efficiency and providing insights for the development of novel vaccines and therapeutic interventions.
